# A qualitative analysis of immigrant population health practices in the Girona Healthcare Region

**DOI:** 10.1186/1471-2458-10-379

**Published:** 2010-06-29

**Authors:** C Saurina, L Vall-llosera, M Saez

**Affiliations:** 1 Research Group on Statistics, Applied Economics and Health (GRECS), University of Girona, Campus de Montilivi, Girona 17071, Spain; 2 CIBER of Epidemiology and Public Health (CIBERESP), Biomedical Research Park, Doctor Aiguader 88, Barcelona 8003, Spain

## Abstract

**Background:**

The research we present here forms part of a two-phase project - one quantitative and the other qualitative - assessing the use of primary health care services. This paper presents the qualitative phase of said research, which is aimed at ascertaining the needs, beliefs, barriers to access and health practices of the immigrant population in comparison with the native population, as well as the perceptions of healthcare professionals. Moroccan and sub-Saharan were the immigrants to who the qualitative phase was specifically addressed. The aims of this paper are as follows: to analyse any possible implications of family organisation in the health practices of the immigrant population; to ascertain social practices relating to illness; to understand the significances of sexual and reproductive health practices; and to ascertain the ideas and perceptions of immigrants, local people and professionals regarding health and the health system.

**Methods:**

Qualitative research based on discursive analysis. Data gathering techniques consisted of discussion groups with health system users and semi-structured individual interviews with healthcare professionals. The sample was taken from the Basic Healthcare Areas of Salt and Banyoles (belonging to the Girona Healthcare Region), the discussion groups being comprised of (a) 6 immigrant Moroccan women, (b) 7 immigrant sub-Saharan African women and (c) 6 immigrant and native population men (2 native men, 2 Moroccan men and 2 sub-Saharan men); and the semi-structured interviews being conducted with the following healthcare professionals: (a) 3 gynaecologists, (b) 3 nurses and 1 administrative staff.

**Results:**

Use of the healthcare system is linked to the perception of not being well, knowledge of the healthcare system, length of time resident in Spain and interiorization of traditional Western medicine as a cure mechanism. The divergences found among the groups of immigrants, local people and healthcare professionals with regard to healthcare education, use of the healthcare service, sexual and reproductive healthcare and reticence with regard to being attended by healthcare personnel of the opposite sex demonstrate a need to work with the immigrant population as a heterogeneous group.

**Conclusions:**

The results we have obtained support the idea that feeling unwell is a psycho-social process, as it takes place within a specific socio-cultural situation and spans a range of beliefs, perceptions and ideas regarding symptomology and how to treat it.

## Background

The phenomenon of people migrating from developing countries and/or countries in more precarious political/economic situations is a process of great relevance in Europe. In Spain this process assumes great importance due to it being a relatively recent phenomenon that poses new challenges with regard to the capacity of the healthcare services aimed at satisfying these new emerging requirements [1].

According to data from the European Statistics Office, Spain was the EU country that received the highest number of immigrants in absolute terms in 2005: 652,300 people, representing almost all (1.5%) of the 1.7% growth in the total population [2].

Within Spain, Catalonia, Madrid and Valencia are the autonomous regions which have the largest immigrant populations, comprising over 60% of the total registered immigrant population [3]. Catalonia hosts 20.95% of the immigrant population in Spain, representing 16.3% of the total population of Catalonia, while Girona hosts 13.7% of immigration in Catalonia, which accounts for 20.92% of its population. The African group represents approximately a quarter of the total number of immigrants.

In the case of the Catalan healthcare system, the capacity of the healthcare services to meet new challenges arising from the arrival of new users in diverse situations and with a heterogeneous history and culture is an element of great importance in strategic healthcare planning [1]. Surveys carried out in Catalonia indicate that in order to guide the design and implementation of quality healthcare policies it is necessary to conduct more in-depth research into the knowledge of the immigrant population and integrate a comparison with the native population into said research [4].

The research we present here forms part of a two-phase project - one quantitative and the other qualitative - assessing the use of primary health care services. Moroccan and sub-Saharan were the immigrants to who the qualitative phase was specifically addressed [5]. The objectives of said study included: identifying and characterising the health status of the diverse collective of immigrants residing in Girona, Spain; estimating health needs among the different collectives of immigrants; and studying the behaviour of immigrants as users of primary health care in order to determine the needs of their collective.

During the first phase, i.e. the quantitative phase, of the project, we were be able to ascertain the current use immigrants and the native population make of the healthcare system, both extensively and in a generalised sense for Catalonia as a whole. However, this does not provide us with an in-depth understanding of the discourses that explain some of the results obtained. In fact, it was only the Moroccan and sub-Saharan immigrants whose behaviour differed from that of the native population with regard to some primary health care services, at least from a quantitative point of view.

This paper specifically presents the qualitative phase of said research, which is aimed at ascertaining the needs, beliefs, barriers to access and health practices of the immigrant population in comparison with the native population, as well as the perceptions of healthcare professionals. The specific aims of this paper were: (a) to analyse the possible implications of family organisation on the health practices of the immigrant population; (b) to ascertain social practices with regard to illness; (c) to understand the significances awarded to sexual and reproductive health practices; and (d) to ascertain the ideas and perceptions immigrants, local people and professionals have of health and the health system.

## Methods

### Study design

The qualitative phase of the study was implemented from a discursive perspective, whereby discourses are considered practices that explain social processes [6]. In particular, narrative analysis was used.

The foreign groups object of this study comprise economic immigrants, considered to be those people born in a country which comes under the classification of developing countries proposed by the United Nations Development Program (UNDP) [7,8].

Based on the results obtained in the quantitative phase, six profiles of respondents were considered (see Table 1) (immigrant Moroccan women, immigrant sub-Saharan African women, immigrant and native men, gynaecologists, administrative staff and nurses). The main reasons for this categorisation were: the most notable data and that most divergent from the native population with regard to the use of sexual and reproductive healthcare services was linked to the population with African origins. No significant differences were noted in the use of the healthcare system between the native male population and the immigrant male population included within this quantitative study. There was evidence of the need to broaden the knowledge base with regard to the views of healthcare professionals.

**Table 1:** Sample

Group	Classification	Method	Area
	Moroccan women	Discussion groups	Banyoles
Patients	Sub-Saharan African women	Discussion groups	Banyoles
	Native and immigrant men	Discussion groups	Salt

	Gynaecologist	Interviews	Salt
Healthcare professionals	Administrative staff	Interviews	Salt
	Nurses	Interviews	Salt

Source: own data

The fieldwork was conducted during the months of June and July 2007.

The study was conducted in the Girona Healthcare Region, specifically in the Basic Healthcare Areas of Salt and Banyoles, these being the areas of the Region with the highest percentage of immigrant population [5]. With regard to the discussion groups, in Salt this work was done with (a) mixed groups of immigrant and native population men and in Banyoles it was done with women from (b) Morocco and (c) sub-Saharan Africa. Semi structured interviews with healthcare professionals were conducted at the Martí i Julià Hospital Park in Salt. (See Table 1).

Medical staff, nurses and particularly cultural mediators from the participating Basic Healthcare Areas were responsible for choosing the participants in the discussion groups. Cultural mediators in our country are professionals who collaborate in the care of social needs of immigrant communities and orient their action towards the prevention and resolution of individual conflicts, family or group that occur in the field of health, the education, social welfare and community living. Mediators must have good language skills in both languages, knowledge of the area of health as well as knowledge, skills and attitudes specific intercultural competence. The only exclusion criteria for participating (other than the categorisation criteria) were that subjects had not participated in the quantitative phase of the project in order than they were not conditioned by the answers given on the questions of the quantitative phase of the project and that women were mothers as one of the main aim of the focus group for women was to ascertain their family situation and the situation about children. Finally, nineteen people participate in the discussion groups (13 woman and 6 men). The regions were they are coming from the women were: 6 women from Morocco and 7 women from Gambia. Regions of origin from men were: 2 men from Morocco, 2 men from Gambia and 2 native men In addition, we conducted seven in-depth interviews (1 administrative staff, 3 nurses and 3 physicians).

### Data gathering techniques

Two data gathering techniques were established: (a) discussion groups and (b) in-depth interviews. Discussion groups are debates on a specific theme carried out by groups of people who represent characteristics of relevance to the research [9]. The subsequent debate relates to how meanings are produced and negotiated from everyday interaction between people, allowing us to ascertain what significances the object of study holds for them and how these are reconstructed in social interaction [10]. Furthermore, divergent stances can be found within the group and in comparison with other groups [11], resulting in a group discourse on the ideas, values and perceptions of a specific group (see the discussion guide in Appendix I). The aim of the semistructured individual interviews was to complement the aforementioned data with the views of healthcare professionals and support staff regarding the use of the healthcare system by the immigrant population in comparison with the native population. The broad topics for in-depth interviews were in order to know: the major impact on work by the arrival of immigrant; the perceived attitudes of the patients, the changes observed in recent times in the attitude of patients, the changes in attitude of professionals, the major differences in behaviour between the native and the immigrant population in order to detect de main differences in the quality of service and to gather suggestions for improvement. Both the discussion groups and the interviews were recorded digitally in order to maintain the reliability of the data for its subsequent analysis.

In order to ensure the cultural relevance of study instruments for study participants, before both discussion groups and, to a lesser extent, in the interviews, we carried out a pilot test with the cultural mediators from the centres as participants.

### Analysis of the data

Analysis of the discursive data began with the literal transcription of all oral content obtained from the discussion groups and interviews. Following this, all paper and oral materials were compared in order to ascertain the degree of consistency between them and guarantee the reliability of the analysed data [12]. Lastly, we eliminated any data that might reveal the identity of the participants.

The discursive analysis of the data comprised the following steps: 1. Dividing the content of the discussion groups into four axes - a) what is known about the family situation, b) types of behaviour when facing illness, c) sexual and reproductive health, and d) perception of treatment received at health centres - and dividing interviews according to these axes - a) the sexual and reproductive health of users and b) types of patient behaviour when facing illness. 2. Identifying units of significance, in this case sentences containing elements of relevance to the analysis. 3. Maintaining the context in which the sentence was constructed, identifying the units of significance within the original text, allowing the movement back and forth between the systematised content and the original text in order to guarantee that the analysis reflects what the subjects meant to say. 4. Creating analytical categories from the axes and the units of significance. 5. Establishing associations between the axes and the categories according to the groups in the sample. 6. Establishing discursive disparities and similarities between each group. 7. Integrating the discursive similarities, disparities and associations between the axes and categories into one single analytical text. 8. Using the Atlas Ti computer program [13] to produce a conceptual map for integrating the axes and factors related to the understanding of the role of healthcare system user among the immigrant population into a complex visual network. 9. Drawing up results.

All information obtained was worked on by two independent groups of researchers in order to achieve a set of consensual data. This was then interpreted in participative work sessions with cultural mediators for each specific group of immigrants.

### Ethical aspects

The study has been evaluated and approved by the Girona Municipal Health Care Institute's Ethical Committee for Clinical Research (CEIC-IAS), which complies with the European Medicines Agency's Guidelines for Good Clinical Practice, CMPMP/ICH/135/95, reference number S041-386. Only the investigators and monitors/auditors working on the study will have access to the data of subjects who agreed to participate.

## Results

**Figure 1:**
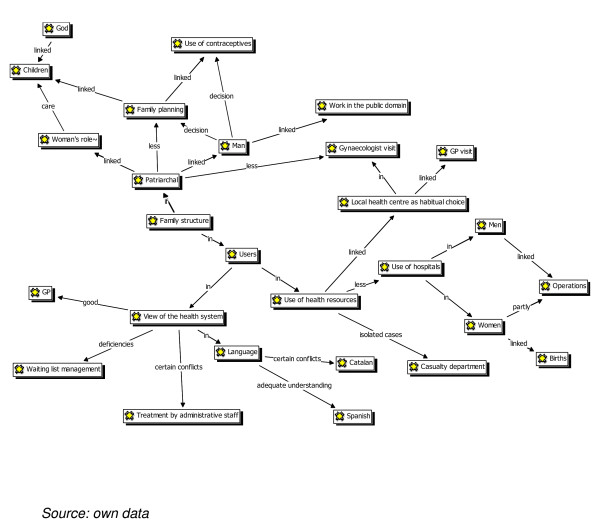
***Atlas-Ti: *Users'views**.

Users' views of the healthcare system (see Figure 1: atlas-ti):

Figure 1 shows the four areas, namely: a) what is known about the family situation, b) types of behaviour when facing illness, c) sexual and reproductive health, and d) perception of treatment received at health centres.

Categories were established and each of the axes related to the analytical categories that reproduce the meaning units. Partnerships were established between these axes and the categories according to different groups of the sample. Finally, the differences and discursive similarities were established between each group and the differences, similarities and associations integrated into a single concept map.

### Gender roles within the family

The roles of women differ in the groups of immigrants studied. This is clearly reflected in caring for small children, with the woman assuming the "emotional role" of caring for the family, the children and the home, and, according to the women interviewed, the man assuming the role of provider of material (economic) support.

Woman from Morocco: *"At the moment I don't want (more children), later I will have one and that's it, no want more. Yes, it's that many children here has a high cost and one person alone (the husband) can't do anything (...)"*

Women from Morocco consider having more than one child as desirable, the responsibility of caring for them is identified as an element which restricts them in establishing links to the public domain, in particular the labour market. Ultimately, the importance of "going out" into the public domain, establishing relationships outside the close environment of the family and economic stability assume different meanings depending on the group studied.

In the group of women from Gambia, the roles of the man-woman couple are clearly differentiated, according to the women, the man takes decisions on economic aspects and family planning, and the woman is in charge of caring for the children and the home. The women from the Moroccan discussion group award less importance to the role of the woman as being the one responsible for looking after the home and children.

Woman from Gambia: "*They don't help much. Because he has to work (the husband). If you have a lot of children you don't go to work and you stay at home to look after children (...) he can't come and leave his work" (...)*.

Woman from Morocco: "*I prepare the food and he washes the dishes, I don't know, he helps. There are days when I have a lot of work and he looks after the children, changes their clothes, helps at home, I don't know" (...)*.

### Family planning (sexual and reproductive health)

In the case of women from Gambia, the gender of the gynaecologist is particularly relevant as it is placed above the role of doctor: the women from the Gambian discussion group say that they do not go to male gynaecologists for check-ups but they do go to females. Furthermore, they say they are aware of methods of contraception but do not use them, arguing that most women in their country do not. Finally, and again according to them, it tends to be the husband who takes the decision on whether to use contraception or not and the number of children the family will have.

*"Yes, if husband wants... take, if no want, no*"

"If he decides you take it so you don't have every year, but mine no"

With regard to the women from the Moroccan discussion group, differences were detected in terms of practices of caring for sexual and reproductive health depending on where they came from (village or city, north or south of the country) and the generation.

Woman from Morocco: *"It depends, rural people have about five or six" *[children] *(...)*.

(our comments appear in brackets).

Woman from Morocco: *"Yes. Before, grandparents and a lot more children, ten, eleven" (...)*.

People from villages located a long way from cities and older generations (over 40 years of age) have similar conceptions to those expressed by the Gambian women with regard to sexual and reproductive health. In one case, a 50 year-old woman did not want to be attended to by a male gynaecologist despite the fact that her health was possibly at risk.

*"I know a woman, she didn't want to come to the gynaecologist for that, I want a woman or I don't want a gynaecologist, and in the end she didn't go and it was very bad for her, that woman (...)*"

The view of the women in the Moroccan discussion group, between 20 and 30 years of age, was similar to the Western view with regard to taking care of your body and family planning (they had between 2 and 3 children, by contrast with the women from Gambia, who had between 5 and 8 children), and also considered possible psycho-social, economic and health consequences from a lack of family planning. In this respect, reference was made to limitations in being available to work due to caring for children, economic costs, implications for health and psycho-social consequences, relating "doing things" to going out into the public domain.

"I'm not going to stay at home all the time doing nothing"

"I know women who have more than three children and is not healthy"

### Use of health resources

Generally speaking, knowledge and normalised use (according to European Western cultural standards) [14] was observed of the functions in primary healthcare, hospital attendance and hospital Casualty attendance.

They say that in most cases they use the primary health centre, and that they first schedule a visit and then go to see their GP. They identify this service as being the one they are most comfortable with, arguing that there is greater trust and more monitoring of the patient's case history. These elements assume particular relevance in the choice of this as the first resource and as the "appropriate mechanism" in cases where there is no urgent need.

Woman from subsaharan Africa: *"My doctor, her... Maite,...' *[the name of her doctor] '... *very good. What she say me now, do you want to change doctors?' *[in response to the possibility to change her doctor] *'...But I am happy with her"*

(our comments appear in brackets).

Native man: *"If I'm not well...' *[If I am sick] '... *it hasn't happened very often, I come to see my GP" *[the family doctor]

(our comments appear in brackets).

Both immigrant and native users evaluate the hospital Casualty service as being distant. They feel anonymous and say they use the Casualty service in extreme cases and hospital attendance when sent by the GP due to a specific need.

Immigrant man: *"well in my case, as I am tough, no (ha ha). I don't need to, no...for a temperature or something like that, it's gone the next day. I mean, if you see it's an illness and you have to go to see the doctor and it really isn't going to go away ... three, four days of temperature. (...) yes. The truth is that when it looks serious I go to Casualty because if I don't I'll end up by the side of the road!"*

### Views of the healthcare system

Both the immigrant and native population express satisfaction with the primary healthcare service, their being some complaints with regard to the coordination of the administrative services.

Native man: *"I had a doctor's appointment at 11.15 and the x-rays at 10.20, it got to 11, there was nobody and I was waiting there on my own and I went there and they said no, they'll call you and then I see they were coming from having breakfast outside and they still took a while to call me, and that shouldn't happen. Sometimes, as we know, it depends on the staff on duty. Sometimes I have gone, the other day I went, I had an appointment at 11.20 and it was 11 and they took me straight away because of the girl, but sometimes there are people that..."*

In some cases the immigrant groups referred to the language as a factor which made fluid interaction difficult. If the foreigner did not know any of the official languages of Catalonia, they developed a series of communicative strategies which helped them to interact with the doctor. The most notable of these were: (a) being accompanied by someone close who knows the Catalan healthcare system codes and language, (b) mediators at the health centre as a communicative link on a language level and (c) use of an unofficial language known by healthcare professionals and users (in these cases, English) as a "communicative bridge".

Immigrant man: *"Well... I speaking English look for someone to speak English to act as intermediator"*

With regard to the hospital healthcare service, both women and men from the different groups participating in the study expressed discontent with regard to the wait to receive attention from specialists. Furthermore, according to those interviewed, this discontent is also shared by the medical specialists themselves.

Immigrant man: *"I waited a year. The doctor told me they had to operate and I asked him how long I had to wait and he said 9 months to a year, well in that case put me on the list because when I can no longer walk... waiting a year in a place... and after a year they operated on me on 3rd December and on 7th it was a year" 3 *(see Table 1).

Immigrant man: *"Yes, the waiting lists. The other day here with X - the GP - there was a man, he was Moroccan too, it seems he needed something urgent and he goes downstairs and I was here, and he calls, he comes and he says, downstairs they are giving me an appointment for October and X got angry, came downstairs and said 'Hey, if I put urgent, why are you giving me October or a non-urgent appointment? and he got angry here and the last thing I heard X say to them was 'Well then send them all to Casualty and that's how we get it done quickly, right?'"*

### The views of healthcare professionals (see Figure 2, Atlas-ti)

**Figure 2:**
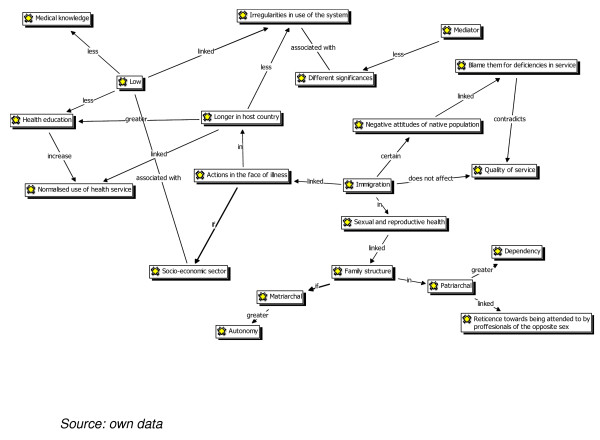
***Atlas-Ti: *Healthcare professionals'view**.

Figure 2 shows the two main areas, namely: a) the sexual and reproductive health of users and b) types of patient behaviour when facing illness. As with Figure 1, categories were established and each of the axes related to the analytical categories that reproduce the meaning units. The differences and discursive similarities were established between each group and the differences, similarities and associations integrated into a single concept map.

### Sexual and reproductive health

There is a noticeable difference between the views of the professionals and the Moroccan women interviewed with regard to the attitude of users towards medical attention from the opposite sex.

In the experience of healthcare system professionals, men and women from this culture have difficulties undressing in front of a doctor or nurse of the opposite sex.

This is particularly true for gynaecologists:

Administrative staff: *"Moroccans are the ones who most commonly ask to have a female gynaecologist"*

Furthermore, they also say that Moroccan women tend to come accompanied by female or male friends when they have to visit the gynaecologist.

### Types of behaviour in the face of illness

According to healthcare professionals, one of the largest problems in the relationship between the doctor and the immigrant patient is communication, both on the level of language and also, particularly, on the level of different significances with regard to health-illness and healthcare attention.

Nurse: *"We go to the doctor straight away, they don't come unless it's very serious"*

Healthcare professionals distinguish between different practices in the use of the healthcare system according to the group of users. Generally speaking, they say that use of the healthcare system is more irregular.

Gynaecologist: *"you have to really stress to Moroccan users that they come to the health centre when they have an appointment, they often don't come, with sub-Saharan patients you have to remind them to be punctual" (...) "with South American patients there is no problem with the language but they demand much more. They are patients with a higher cultural level who do not accept the word of the professional so submissively; they demand more*"

Administrative staff: *"over there, in their country you have to pay for healthcare and when they come here they're not used to going" (...)*

The use of medical assistance under equal conditions is greater among native subjects and the immigrant population that has resided longer in Spain. One explanation for these divergences is a greater knowledge of the resources available, but the main reason is the high degree of interiorisation of the concept of caring for the body and considering medicine to be a legitimate element that provides said care in our culture [14].

Although discourses and perceptions regarding the patient being unwell are different, healthcare personnel tend to regard "legitimate knowledge" as scientific knowledge, considering everyday knowledge or other healthcare mechanisms not part of the medical system as incorrect.

Gynaecologist: *"you attend them from a Western viewpoint and I think that is how it has to be"*

They also argue that most immigrants belong to a low social stratum and lack knowledge with regard to medicine.

There is emphasis of the importance of the role of cultural mediator as a facilitator of communication between immigrant users and healthcare personnel.

Administrative staff: *"Immigrants generally understand the administrative processes but they need help on a communicative level."*

Finally, with regard to the factor of diverging significances between health and illness and healthcare, a positive factor in achieving a normalised use of the healthcare system is noted as being the length of time that the patient has been in the host country, as this facilitates the patient's adaptation, comprehension and relationship with the healthcare personnel. In other words, the management of common significances between the host population and immigrants. In particular, it allows for a change of attitudes and beliefs with regard to health, illness and especially in matters referring to sexual and reproductive health.

Gynaecologist: *"immigrant women are now starting to have tubal ligation, they are also taking oral contraceptives or using the IUD" (...) they also understand that children are more expensive to maintain here"*.

A resume of the major themes that emerged from the interviews and focus groups among the participants, is shown in Table 2.

**Table 2:** Frequency (%) of responses among participants

Major themes	Type of participant and their view	%
Gender roles within the family	Women from Morocco	
	*Western view of gender roles*	47
	
	Women from Gambia	
	*Traditional view of gender roles*	53

Family planning	Women from Morocco	
	*Western view in young urban women*	85
	
	Women from Gambia	
	*Traditional view in all women*	95
	
	Health care professionals	
	*Traditional view in Gambian and Moroccan women*	90

*Use of health resources*	African men	
	*Adequate knowledge of channels for using the health system*	67
	
	Native men	
	*Adequate knowledge of channels for using the health system*	100
	
	Health care professionals	
	*Immigrant population uses primary care services in more extreme situations than native population*	70
	
	Moroccan women	
	*Adequate knowledge of channels for using the health system*	80
	
	Gambian women	
	*Adequate knowledge of channels for using the health system*	40

*Views of the healthcare system*	African men	
	*Excessive waiting time for medical specialist*	95
	
	Native men	
	*Excessive waiting time for medical specialist*	95
	
	Health care professionals	
	*Immigrants are not punctual for medical appointments*	80
	
	Moroccan women	
	*Excessive waiting time for medical specialist*	95
	*Catalan language problems*	15
	
	Gambian women	
	*Excessive waiting time for medical specialist*	95

Source: own data

## Discussion

Generally speaking, knowledge and normalised use (according to European Western cultural standards) [15] was observed of the functions in primary healthcare, hospital attendance and hospital Casualty attendance. Knowledge of the healthcare system and norms of use in this country do not always coincide, and this becomes evident in the practices of those hailing from other cultures.

Firstly, current changes in the family structure and ways of awarding significance to gender roles are reflected in the discourses of those participating in the study with regard to the organisation of the family [16]; there is a noticeable difference in the significance awarded to caring for one's body, the customisation of this and the repercussions on one's life and family planning among the women interviewed. There is evidence then, that although in Western culture the changes and questioning of the female role are in full swing, questioning the patriarchal ideology that establishes the man as the person with most authority, who provides economic support and a link to the public domain, and the woman as the person responsible for the emotional domain, caring for the family and closely linked to the private domain, the questioning of the traditional solid, patriarchal and extended family does not occur (or not equally) in all cultures and situations [17].

Secondly, with regard to perceptions of the specialised healthcare service, the urgency attached to not feeling well and the subsequent search for treatment, in addition to the perception of time as a negative factor in the course of untreated illness, contrast with the real waiting time to be attended by a specialist [18]. In respect of this, both the men and women interviewed expressed discontent with the length of the wait to see a specialist. This discontent is reflected in the discourses of the native population [19], immigrants and according to the interviewees, also the medical professionals themselves. When making an appointment to see a specialist, the divergence between what the GP says (a figure of great trust and authority for the patient) and what those who schedule the appointments say, expressed openly before the patient, serve to increase the feeling of urgency and prevailing need to be attended. Therefore, if expectations are not fulfilled, the feeling of not being well increases.

Thirdly, a group's origins cannot be relied on to determine shared significances with regard to (in our case) sexual and reproductive health, but rather it is personal experiences arising from the interaction between subjects in a more local context that determines the ideas and practices of daily life [20].

This work could have some limitations. Firstly, it is a local study, at least from a geographical point of view. In addition, it derives from a quantitative study. In fact, as we point out above, it corresponds to the qualitative phase of a project carried out immediately after the quantitative phase. In our current and future research we would like to prioritise the qualitative perspective and use the possible results to guide the quantitative phase. Furthermore, our current research has been extended to all of Catalonia, to other health care services, emergency services in particular, and to other immigrant subgroups, including Latin Americans, East Europeans (non EU) and Asians (Chinese and Pakistanis).

## Conclusions

The results we have obtained support the idea proposed by Mechanic [21]: feeling unwell is a psycho-social process, as it takes place within a specific socio-cultural situation and spans a range of beliefs, perceptions and ideas regarding symptomology and how to treat it. Specifically, family roles have implications for health practice because it is women who care for the health of children. The perception of one's own health is a reflection of the sociocultural situation of the patient. Sexual and reproductive health practices are linked to the origin of the patient. There was no difference in perception of the health care services between the groups analysed.

## Competing interests

The authors declare that they have no competing interests.

## Authors' contributions

All authors:

1) Have made substantial contributions to conception and design, or acquisition of data, or analysis and interpretation of data;

2) Have been involved in drafting the manuscript or revising it critically for important intellectual content; and

3) Have given final approval of the version to be published.

## Appendix I. Discussion guide

### FOCUS GROUP 1.- Immigrant and native men

**Aims: **Ascertain differences in self-perception of health.

Ways of behaving towards illness.

Reasons for hospitalisation.

Situation with regard to health check-ups.

Perception of treatment received.

We are conducting a study to help us determine whether there are differences in the state of health of the different collectives who use the health services and the use they make of them. Our aim is to gain an understanding of the reality facing the current public health system in order to be able to suggest improvements for it.

### Open questions asked

1.- For what reasons do you go to the doctor's? With what symptoms? How often ?

When you do go, where do you go? - To the local health centre, the hospital... why?

2.- Who do you turn to if you have any questions about your health?

(Ascertain exactly what they understand by health)

Have you heard of cultural mediators?

3.- Have you or any family member had to be hospitalised?

Who? Why? How long for?

4.- Do you have regular health check-ups? Why?

Who decides this? You yourself? The company? Do you not have them?

5.- In general, when you have had a health problem, how did you find the service you received?

Do you feel you were treated well by the medical staff?

Did they take your culture into account? Do you think that they treat you differently according to who you are? In what respect?

What is the most important aspect of the service for you?: (accessibility, equality, how well they resolve your problem, additional tests)

What's the most important quality of the doctor? (resolving your problem, capability, empathy, respectfulness). Is the gender of the doctor important

6.- What do you think is the most important thing for improving the health care system ?

7.- Would you like to add anything else?

### FOCUS GROUP 2.- Immigrant women

**Aims: **Ascertain family situation: children, reuniting with other members.

Ways of behaving towards illness.

Reasons for hospitalisation.

Sexual and reproductive health

Perceptions of treatment received.

We are conducting a study to help us determine whether there are differences in the state of health of the different collectives who use the health services and the use they make of them. Our aim is to gain an understanding of the reality facing the current public health system in order to be able to suggest improvements for it.

### Introduction

Name, age, country of origin and date they left, how long they have lived in Banyoles, marital status, number of children they are responsible for, work outside the home.

### Open questions asked

1.- What process did your arrival here take? Who left first? How did the family reunite here? If you have children, where are they? If they are in your home country, who cares for them now? Do you plan to return to your country of origin? And your children?

2.- Who cares for the children on a day-to-day basis? School, doctor, homework, habits,.... What do they do when they are ill? Who takes them to the doctor? Where do they go? (what type of illnesses are we talking about here?)

3.- As for you, who do you turn to if you have any questions about your health?

For what reasons do you go to the doctor's? With what symptoms? How often?

When you do go, where do you go? - To the local health centre, the hospital... why?

(Ascertain exactly what they understand by health)

Have you heard of cultural mediators?

4.- Do you use any family planning methods? Do you know different methods? Where from? Who takes the decision? You yourself, together with your husband,...

5.- Do you go to the gynaecologist for check-ups? When? Do you visit the doctor for check-ups when you are pregnant? Do you prepare yourselves for the birth? Where? (Health centre, home)

6.- Have you or any family member had to be hospitalised?

Who? Why? How long for?

7.- In general, when you have had a health problem, how did you find the service you received?

Do you feel you were treated well by the medical staff?

Did they take your culture into account? Do you think that they treat you differently according to who you are? In what respect?

What is the most important aspect of the service for you?: (accessibility, equality, how well they resolve your problem, additional tests)

What's the most important quality of the doctor? (resolving your problem, capability, empathy, respectfulness). Is the gender of the doctor important?

8.- What do you think is the most important thing for improving the health care system ?

9.- Would you like to add anything else?

## Pre-publication history

The pre-publication history for this paper can be accessed here:

http://www.biomedcentral.com/1471-2458/10/379/prepub
